# A Facile Strategy to Fabricate Antistatic Polyamide 1012/Multi-Walled Carbon Nanotube Pipes for Fuel Delivery Applications

**DOI:** 10.3390/polym12081797

**Published:** 2020-08-11

**Authors:** Wanli Li, Lili Wang, Xia Dong, Dujin Wang

**Affiliations:** 1 Institute of Systems Engineering, Academy of Military Sciences, Beijing 102300, China; 13520351817@139.com; 2 State Key Laboratory of Bio-Fibers and Eco-Textiles, Collaborative Innovation Center of Marine Biobased Fiber and Ecological Textile Technology, Institute of Marine Biobased Materials, Qingdao University, Qingdao 266071, China; llwang@qdu.edu.cn; 3 Key Laboratory of Engineering Plastics, Beijing National Laboratory for Molecular Sciences, Institute of Chemistry, Chinese Academy of Sciences, Beijing 100190, China; djwang@iccas.ac.cn

**Keywords:** PA1012/MWCNTs, fuel pipes, conductivity

## Abstract

Developing antistatic long chain polyamide (LCPA) resins and fabricating the corresponding fuel pipes are challenges but necessary. Herein, a facile but effective strategy was put forward to fabricate LCPA resins with a superior conductivity, meeting the requirements of electrostatic sub-conductors. The strategy was based on, first, the incorporation of a large amount (15 wt%) of multi-walled carbon nanotubes (MWCNTs) into a polyamide 1012 (PA1012) matrix as a master batch, which formed a dense conductive network. Subsequently, it was diluted with PA1012 granules to produce base resins, and the reprocessed nanocomposites with a critical content of MWCNTs (3 wt%) could generate an effectively interconnected conductive network, with sparse and thinning features. Using the base resins, fuel pipes for automobiles, petrol stations and high pressure applications were successfully fabricated, where the thin conductive network was transformed into a thick one due to external field-induced re-agglomeration of MWCNTs. In this way, the obtained fuel pipes combined excellent conductive and barrier properties, and mechanical properties at high and low temperatures. These comprehensive properties also arose from the uniform dispersion of MWCNTs in an LCPA matrix, even without coupling agents; the attractive interaction between MWCNTs and the polyamide chains contributed to their strong interface adhesion. Thus, this research provides a versatile approach to fabricating antistatic LCPA resins, which will certainly extend their application to vehicle fuel systems.

## 1. Introduction

An aliphatic long chain polyamide (LCPA) is characterized by more than ten methylene units between adjacent amide groups. Contributed by flexible methylene blocks and polar amide groups, an LCPA has superior mechanical performance, chemical solvents and fuel resistance, and dimensional stability; they have been widely applied in the fields of automobile manufacturing, electronic appliances and the commodity market, particularly for vehicle fuel pipes and air brake pipes [1,2,3,4,5]. However, a prerequisite LCPA used in the application of fuel pipes must have excellent antistatic properties [6,7]. Because it is driven by a fuel pump, gasoline flows at a high speed through pipelines between a fuel tank and engine. Under this condition, friction between the gasoline and the inner wall of the pipes may produce electrostatic charges, which may cause a serious electrostatic accident during accumulation. Thus, improving the conductivity of electrically insulating LCPA, without sacrificing its mechanical properties, is necessary to broaden its applications in vehicle fuel systems.

In order to achieve a conductive polyamide, several methods have been adopted in recent years, which can be classified into two types according to the categories of antistatic agents. First, conductive nanoparticles [8,9], including carbon nanotubes [10,11,12,13], graphene [14,15,16,17,18] and carbon black [19,20,21,22], have been popular for use in fabricating polyamide-based nanocomposites. Here, enhancing the interfacial adhesion between nanopowders and the corresponding matrix has attracted a great deal of attention as it determines the ultimate properties of the composites. For example, in order to increase the interface attraction between carbon nanotubes and a polyamide matrix, single wall carbon nanotubes (SWCNT) were functionalized with −(CH_2_)*_n_*COCl on their sidewalls, which could covalently link with the alkyl segments of nylon [10]. Haddon et al. also reported a process to engineer an interface between SWCNT and nylon by creating amide groups on the sidewalls of SWCNTs (SWCNT-CONH_2_) [13]. After the incorporation of conductive nanoparticles, the rearranged conductive network endowed polyamide with conductive properties. Second, some intrinsic conductive polymers, which share excellent conductivity due to a particular structure to produce the charge carrier (such as polyaniline [23,24,25,26] and polypyrrole [27,28,29,30]), can impart polyamide with outstanding electrical properties. Dong et al. adopted in situ polymerization methods to synthesize polyaniline powders on the surface of long chain polyamides, which exhibited a conductivity of about 10^−5^–10^−3^ S/cm [24]. The methods mentioned above successfully provide a combination of electrical and mechanical properties for polyamide, but the complicated procedures tremendously hinder their further application in the mass production of fuel pipes. Therefore, developing a facile strategy to fabricate antistatic nylon resins for fuel pipes remains challenging.

In this research, in order to fabricate antistatic LCPA without sacrificing its superior mechanical properties, multi-walled carbon nanotubes (MWCNTs) were selected as antistatic agents. Here, bio-fermented LCPA, polyamide 1012 (PA1012), was used due to its green synthesis procedures [30,31,32]. In order to avoid the aggregation of MWCNTs, PA1012 nanocomposites with a high content of MWCNTs (M = 15 wt%) were firstly fabricated as a master batch, and were subsequently diluted with neat PA1012 granules to obtain a final antistatic PA1012 base resin (M = 3 wt%). Based on the antistatic LCPA base resins, fuel pipes for automobiles, petrol stations and for high pressure applications were successfully produced. The comprehensive properties of fuel pipes, including their electrical properties, barrier properties and mechanical properties, were investigated. By correlating the microstructures, the corresponding mechanism of the antistatic properties of PA1012/MWCNTs was put forward.

## 2. Materials and Methods

### 2.1. Materials

The raw materials of PA1012 resin (−[NH(CH_2_)_10_NHCO(CH_2_)_10_CO]_n_−) were synthesized using bio-fermentation sources, which were kindly supplied by Shandong Guangyin New Materials Co., Ltd (Zibo, China). The melting temperature (T_m_) of PA1012 was about 189 °C and the melting index was 1.39 g/10 min, which was determined using a Melt Flow Indexer at 190 °C according to ASTM D1238 (2.16 kg). Under vacuum conditions, the PA1012 granules were dried at 95 °C for 15 h. The multi-walled carbon nanotubes (MWCNTs, NC7000), with an average diameter of 9.5 nm, average length of 1.5 μm and specific surface area of 250–300 m^2^/g, were produced by Nanocyl sa. Carbon black (CB, Printex L6), with an average diameter of 18 nm and specific surface area of 250 m^2^/g, was produced by Evonik Industries AG (Shanghai, China).

### 2.2. Preparation of Antistatic Resin Based on MWCNTs and CBs

PA1012/MWCNTs blends with a weight ratio of 85/15 were prepared using a 35# twin-screw extruder (screw diameter = 35.6 mm, screw ratio = 44, screw speed = 180 r/min, temperature (T) profiles of 35# in Table 1), and then extruded, granulated and dried. In this way, an antistatic master batch with 15 wt% MWCNTs was obtained. Next, the prepared master batch with 15 wt% MWCNTs was blended with neat PA1012 granules using a 56# twin-screw extruder (screw diameter = 56 mm, screw ratio = 40, screw speed = 180 r/min, temperature profiles of 56# in Table 1), and the same processes mentioned above was performed. Through varying the weight ratio of the master batch and neat PA1012 granules, antistatic PA1012 granules with MWCNTs contents (M) of 1, 2 and 3 wt% were obtained.

PA1012/CBs blends with a weight ratio of 80/20 were prepared using a twin-screw extruder and then extruded, granulated and dried. In this way, a master batch with 20 wt% CBs was obtained, and the processing parameters were similar with those for the MWCNTs.

### 2.3. Fabrication of PA1012 Fuel Pipes

**Figure 1:**
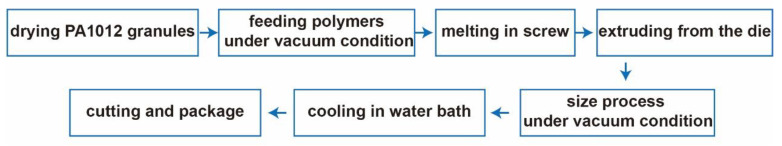
The procedures of fabricating fuel pipes for automobiles.

Based on the antistatic PA1012/MWCNTs granules, three kinds of fuel pipes, fuel pipes for automobiles, for petrol stations and for high pressure applications, were successfully fabricated using extruding techniques. Fuel pipes for automobiles (outer diameter = 6 mm, inner diameter = 4 mm, wall thickness = 1 mm) were fabricated using a 45# single-screw extruder (screw diameter = 45 mm, screw ratio = 30, screw speed = 230 r/min, temperature profiles of 45# in Table 2) in terms of the procedures shown in Figure 1. Similarly, fuel pipes for petrol stations (outer diameter = 63 mm, inner diameter = 56 mm, wall thickness = 3.5 mm) were fabricated using a 55# single-screw extruder (screw diameter = 55 mm, screw ratio = 28, screw speed = 27 r/min, temperature profiles of 55# in Table 2). However, the fuel pipes used in high pressure applications (outer diameter = 166 mm, inner diameter = 146 mm, wall thickness = 10 mm) were produced using a multilayer extruding process, which was composed of an inner PA1012 layer, a steel wire spiral layer and an external polyethylene (PE) layer. The inner PA1012 pipes were fabricated using a 90# single-screw extruder (screw diameter = 90 mm, screw ratio = 25, screw speed = 20 r/min, temperature profiles of 90# in Table 2).

### 2.4. Morphology Analysis

Using scanning electron microscopy (SEM, JSM-6700F, JEOL, Akishima-shi, Japan), the morphology of the as-received MWCNTs, the cross-section surfaces of antistatic PA1012 granules and corresponding fuel pipes, the outside and internal surfaces of the fuel pipes that were coated with a gold layer was collected. Here, the cross-section of materials was obtained by quenching the sample in liquid nitrogen for about 5 min, and then breaking it into two parts along the lengthwise direction.

### 2.5. Surface Resistivity Measurements

According to the standard methods of testing bulk and surface resistivity for solid insulating materials (GB/T 1410-2006), surface resistivity (ρ_s_) was obtained using a resistance meter (ZC46A, Shanghai, China). All tests were repeated at least three times, and the average value was used.

### 2.6. Mechanical Measurements

**Figure 2:**
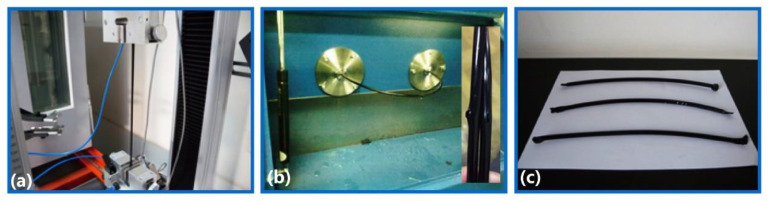
The measurements of (**a**) tensile loading, (**b**) burst pressure and (**c**) fuel permeability for the fuel pipes of automobiles.

The mechanical performance of fuel pipes for automobiles was tested (Figure 2a) using an electronic universal tensile machine (AI-7000M-GD) according to the Chinese standard of multilayer plastic fuel pipes for automobiles (QC/T798-2008). Fuel pipes with a 30-mm length were stretched at a speed of 50 mm/min. The tensile data ranging from −50 °C to 100 °C were first collected at 10 °C intervals. In order to clearly reveal the mechanical transition within the temperature range of 0–10 °C, the tensile data were then collected at 2 °C intervals. Five specimens were tested for each sample, and the average values were adopted for analysis.

According to the same standard rules (QC/T798-2008), burst pressure (P_b_) tests for the three kinds of fuel pipes were also carried out. For example, burst pressure measurements of fuel pipes for automobiles were performed (Figure 2b) using a bursting machine (JR-HT-WA-20). Five specimens were tested for each sample, and the average values were adopted for analysis.

### 2.7. Fuel Permeability Tests

Measuring of fuel permeability (W) for the three kinds of fuel pipes was performed. For example, for fuel pipes for automobiles, one end of the pipe (more than 200 mm in length (L)) was first sealed by melting treatment. Then, 93# gasoline (GB17930) was added inside the pipe and the other end was subsequently sealed. The initial weight (m_0_) and the corresponding weight of the pipe and fuel placed at room temperature for 30 days (m_1_) were collected, and the fuel permeability (W, g/m·d) was calculated based on the weight changes of the pipe (Equation (1), Figure 2c).

W = (m_0_ − m_1_)/30L (1)

## 3. Results and Discussion

### 3.1. Surface Resistivity of PA1012/MWCNTs Nanocomposites

The surface resistivities of PA1012/MWCNTs nanocomposites with various contents of MWCNTs were investigated (Table 3). Obviously, with a MWCNT content of 15 wt%, the surface resistivity (ρ_s_) of the master batch could be less than 10^4^ Ω/sq. When the master batch was diluted with various contents of neat PA1012 granules, the value of ρ_s_ decreased gradually. Specifically, with M = 1 wt%, the surface resistivity of the nanocomposites was about 10^12^ Ω/sq. When M was increased to 2 wt%, the value of ρ_s_ was decreased to about 10^11^ Ω/sq, but the value was unstable in this case. When M was further increased to 3 wt%, the value of ρ_s_ could reach about 10^7^ Ω/sq. Accordingly, after the incorporation of 3 wt% MWCNTs, the surface resistivity of neat PA1012 changed from 10^13^ Ω/sq to 10^7^ Ω/sq, which fully met the requirements of an electrostatic sub-conductor (1 × 10^7^ Ω/sq–10^11^ Ω/sq) and could serve as antistatic LCPA base resins.

In addition, carbon black was also used as an antistatic agent for PA1012, due to its wide range of applications in polymer composites and its low price. It was found that, with a filler content of less than 8 wt%, the surface resistivity of PA1012 varied little, while with more than 10 wt% carbon black, the surface resistivity of the nanocomposites could be acceptable. However, in this case, large amounts of carbon black largely reduced the mechanical parameters, which may be due to serious aggregation of the filler. For example, the elongation at breakage of PA1012 decreased from 331% to 49% after the incorporation of 10 wt% carbon black. Therefore, compared with the carbon black, MWCNTs were the ideal candidates as antistatic agents for LCPA.

**Figure 3:**
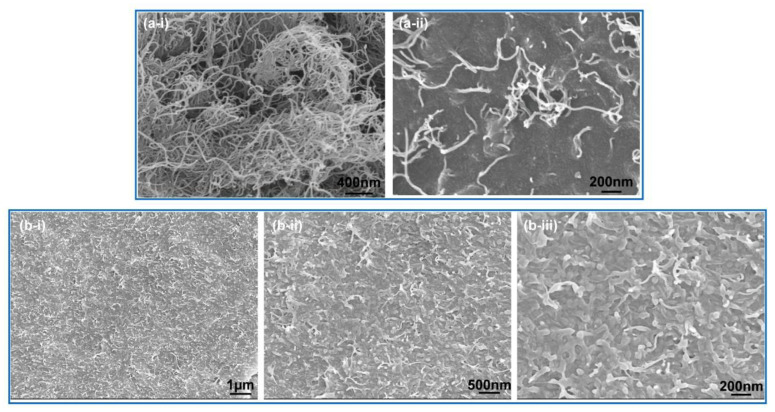
The morphology of MWCNTs (**a**) and the antistatic PA1012 master batch (M = 15 wt%) (**b**).

The morphology of as-received MWCNTs is shown in Figure 3a, where the MWCNTs were dispersed randomly. Figure 3b shows the morphology of a cross-section of the nanocomposites, providing a distribution of MWCNTs in the matrix. Apparently, even with a high MWCNT content of 15 wt%, the antistatic agents were distributed homogeneously without occurrence of aggregation. In general, achieving a uniform dispersion of a large amount of fillers in a matrix is difficult without a coupling agent [33,34,35,36,37,38]. Therefore, without any extra coupling agent, better dispersion of MWCNTs in the PA1012 matrix is a significant achievement in this investigation. In addition, according to the enlarged morphology, the surface adhesion between PA1012 and MWCNTs was strong, and no apparent MWCNTs were pulled out. In conclusion, considering the uniform dispersion of antistatic agents in the matrix and their intimate adhesion, a satisfactory modification method could be expected in PA1012/MWCNTs nanocomposites. Indeed, the MWCNTs could generate an interconnected conductive network and effectively improve the conductivity of PA1012 (Table 3).

### 3.2. PA1012/MWCNT Pipes in Vehicle Fuel Systems

**Figure 4:**
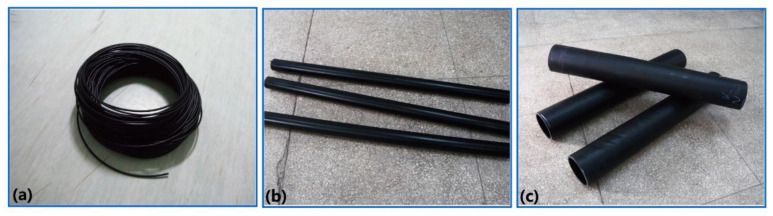
(**a**) Fuel pipes for automobiles, (**b**) fuel pipes for petrol stations and (**c**) fuel pipes used in high pressure applications.

Based on the prepared antistatic LCPA granules (M = 3 wt%), fuel pipes for automobiles, petrol stations and for use in high pressure applications were successfully fabricated (Figure 4), in which the extrusion molding technology and processing stability of large caliber fuel pipes were investigated. The successful fabrication of the three kinds of fuel pipes indicated that PA1012/MWCNTs nanocomposites with M = 3 wt% could serve as base resins for antistatic LCPA pipes, which certainly will extend their application to vehicle fuel systems.

**Figure 5:**
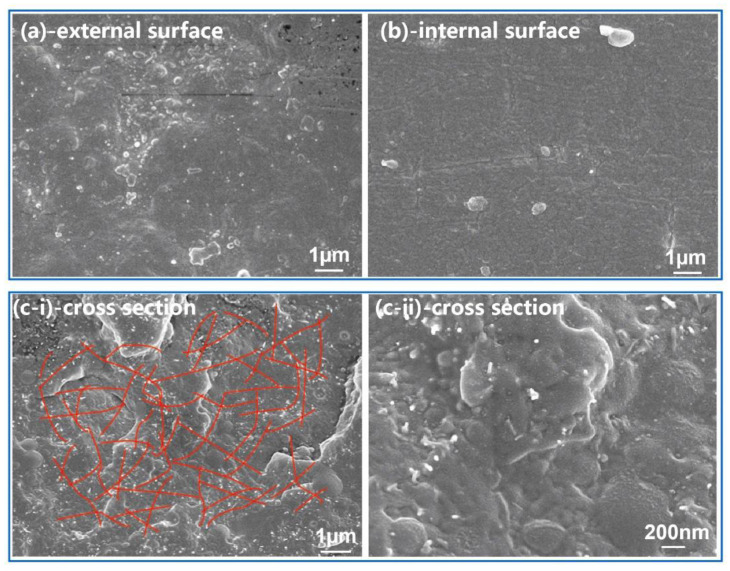
The morphology of the outside and internal surface (**a**,**b**) and cross-section surfaces (**c**) for fuel pipes with 3 wt% MWCNTs.

In order to reveal the dispersion of MWCNTs in the PA1012 matrix after the processing of fuel pipes, the morphology of the fuel pipes was studied from two aspects, where the fuel pipes for automobiles were selected as representative samples. For one aspect, according to the surface of the fuel pipes, both the internal and outside surfaces represented a smooth state, without obvious emergence of MWCNTs (Figure 5a,b). For the other aspect, based on the cross-section surface, the concentration of MWCNTs for the base resin of PA1012 fuel pipes was largely reduced compared with the master batch (Figure 3b). However, even diluted with neat PA1012 granules, the MWCNTs could evenly distribute in the matrix and share a strong interfacial adhesion, which facilitated the construction of a conductive network.

### 3.3. Comprehensive Properties of Fuel Pipes

In order to evaluate the comprehensive properties of the three kinds of fuel pipes fabricated in this research, a series of basic parameters was collected (Table 4). According to the data, the mechanical properties, barrier properties and electrical properties of the three kinds of fuel pipes were superior, and satisfied the requirements of the applications. According to the standard rules QC/T798-2008 “multi-layers plastic tubing for automotive fuel system”, the fuel permeability (W) of fuel pipes for automobiles should be less than 0.05 g/m·d, and the W in our prepared pipes was 0.03 g/m·d, which completely met the requirement of the standard rules. It should be noted that the parameters of the fuel pipes used for automobiles almost reached the international advanced level of similar products.

**Figure 6:**
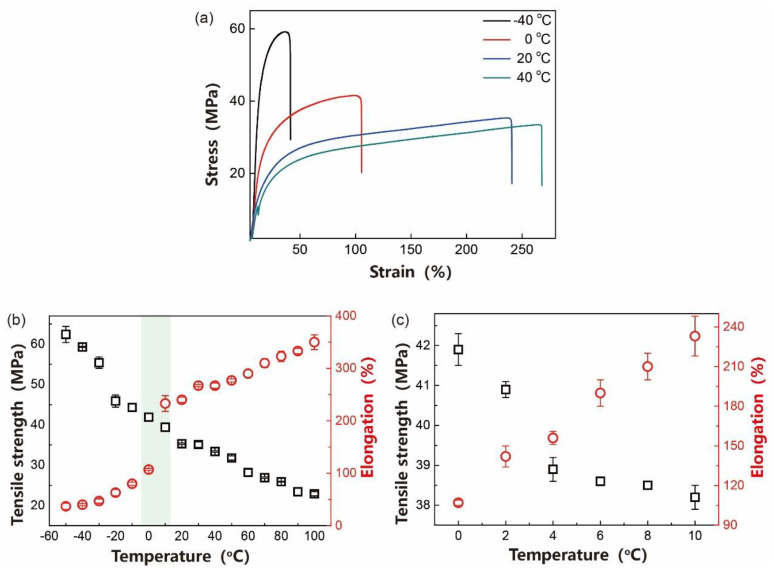
(**a**) The stress strain curves at −40 °C, 0 °C, 20 °C and 40 °C. (**b**,**c**) The temperature dependence of tensile strength and elongation for fuel pipes in the temperature range of −50–100 °C (**b**) and 0–10 °C (**c**).

To further investigate the mechanical properties of the fuel pipes when used at high and low temperatures, the temperature dependence of the tensile strength and elongation at breakage for the fuel pipes was recorded within the temperature region of −50–100 °C (Figure 6); the fuel pipes used for automobiles were selected as representative samples. From the figure, the PA1012 fuel pipes showed a remarkable temperature dependence (Figure 6a) at an ambient temperature (20 °C), the tensile strength of the fuel pipes was 35.3 MPa and the elongation was 240%. With increasing temperature, the tensile strength was gradually reduced, accompanied with an increase in elongation. For example, the tensile strength at 90 °C was 23.4 MPa and the corresponding elongation was 333% (Figure 6b). With a decrease in temperature, the tensile strength could be raised, but reduced elongation occurred. When the temperature was −40 °C, the tensile strength was 59.3 MPa and the elongation was 40%. These variations in mechanical parameters with temperatures were reasonable, and was ascribed to the temperature dependence of the mobility of polyamide chains. From the report of Dong et al., strain-induced crystallization of PA1012 occurred in high temperature regions, which would also contribute to the strength of the composites [39,40]. In summary, although the mechanical parameters of fuel pipes exhibited temperature dependence, both strength and toughness remained superior at extreme conditions, confirming their excellent resistance to high and low temperature conditions. Therefore, these kinds of PA1012 fuel pipes were suitable in automobile applications.

In addition, an apparent transition of elongation could be observed in the temperature region of 0–10 °C; that is, the elongation was abruptly increased with increasing temperature. Herein, the mechanical parameters ranging from 0 °C to 10 °C were collected at 2 °C intervals (Figure 6c) and showed that the elongation was indeed gradually raised with increasing temperature. This may be due to the fact that the mobility of polymer chains became easier at this temperature region and their conformation could be easily adjusted, leading to a high temperature-induced raised elongation. It should be noted that the glass transition temperature (T*g*) of PA1012 was about 40 °C [4], and for PA1012/MWCNTs fuel pipes, the T*g* became small due to the incorporation of extra additives, such as antioxidants.

### 3.4. Mechanism of Antistatic Properties for PA1012 Fuel Pipes

**Figure 7:**
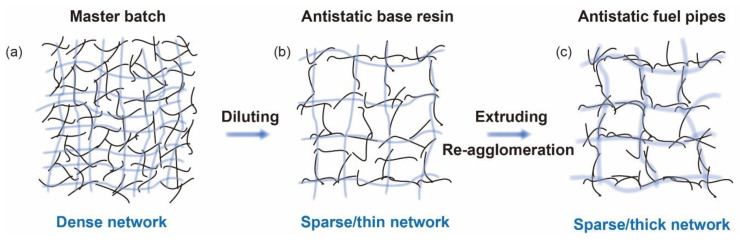
The mechanism of antistatic properties for PA1012 fuel pipes. The image depicted the conductive network of (**a**) master batch, (**b**) antistatic base resin and (**c**) antistatic fuel pipes. (Here the black lines represented the MWCNTs, and the gray blue lines represented the conductive network.).

Based on the electrical and mechanical data, the mechanism of our proposed methods of fabricating PA1012 fuel pipes is illustrated in Figure 7. For the master batch, a large amount of MWNCTs formed a dense conductive network (Figure 7a), while the diluted antistatic base resin (Figure 7b) made the dense conductive network into a sparse one. In the final extruding process, re-agglomeration and orientation of MWCNTs occurred, triggered by external fields, and the thin conductive network was transformed into a thick one (Figure 7c). In this way, fuel pipes with interconnected conductive networks shared superior electrical properties. Additionally, it should be noted that the uniform distribution of MWCNTs in the polyamide matrix, even with high loading, may be due to the attractive interaction between mutual phases, such as hydrogen bonding, whose effect on the ultimate properties of polymers has been pointed out in many systems [41,42,43], and newwork structure forming of long chain polyamides during high temperature processing [44]. The uniform dispersion, the strong interface and the orientation microstructures also gave the nanocomposites a high efficiency of load transfer, improving the mechanical parameters of the materials. Based on the mechanism mentioned above, it could be concluded that in the case of no extra coupling agents, the addition of MWCNTs into a PA1012 matrix enabled it to simultaneously enhance the conductive and mechanical properties, which became suitable materials to fabricate high performance fuel pipes.

## 4. Conclusions

In this research, in order to fabricate the PA1012 base resin of fuel pipes, MWCNTs without any extra coupling agents were selected as the antistatic agent. First, PA1012 nanocomposites with a high content of MWCNTs (M = 15 wt%) were fabricated as the master batch, which were subsequently diluted with the neat PA1012 granules to vary the value of M. It was found that the critical MWCNT content of nanocomposites to form an effectively interconnected conductive network was approximately 3 wt%, and the corresponding nanocomposites could serve as the base resin of fuel pipes. Second, based on the prepared antistatic LCPA resins (M = 3 wt%), the fuel pipes for automobiles, the fuel pipes for petrol stations and the fuel pipes used in high pressure applications were successfully fabricated. The results exhibited that the incorporation of MWCNTs into the PA1012 matrix improved the conductive properties, where the conductive network underwent a dense to sparse and thin, then sparse and thick transition after three processing procedures. Additionally, both the strength and toughness of the fuel pipes remained superior at extreme conditions, confirming their excellent high and low temperature resistance. The superior electrical and mechanical properties arose from the uniform dispersion of MWCNTs in the PA1012 matrix and their strong interface adhesion. Therefore, this research provides a versatile strategy to prepare an antistatic LCPA base resin for fuel pipes, which will definitely extend their applications in vehicle fuel systems.

## Figures and Tables

**Table 1:** Specific temperature of every section for the twin-screw extruding process.

T (°C)	T_1_	T_2_	T_3_	T_4_	T_5_	T_6_	T_7_	T_8_	T_9_	T_10_	T_die_
35#	180	220	230	230	230	220	210	220	230	240	240
56#	180	220	230	230	230	220	210	220	230	240	240

**Table 2:** Specific temperature of every section for the single-screw extruding process.

T (°C)	T_1_	T_2_	T_3_	T_4_	T_5_	T_nose_	T_mold_
45#	180	230	240	240	230	220	
55#	180	190	200	210	/	200	200–175
90#	180	190	200	210	/	200	200–175

**Table 3:** The surface resistivity of PA1012/multi-walled carbon nanotubes (MWCNTs) composites.

Antistatic Agent	MWCNT					Carbon Black		
M (wt%)	0	1	2	3	15	8	10	20
ρ_s_ (Ω/sq)	10^13^	10^12^	≈10^11^	10^7^	<10^4^	10^11^	10^8^	<10^3^

**Table 4:** The basic properties of three kinds of fuel pipes.

Categories of Fuel Pipes	(a)	(b)	(c)
**Applications**	automobiles	petrol station	high pressure
**Inner diameter, mm**	4	56	146
**Wall thickness, mm**	1	3.5	10
**Burst pressure, MPa**	8.48 ± 0.01	3.23 ± 0.12	18.40 ± 0.05
**Fuel permeability, g/m·d**	0.03 ± 0.002	0.11 ± 0.003	0.21 ± 0.009
**Surface resistivity, Ω/sq**	1.1 × 10^7^	1.2 × 10^7^	7.3 × 10^5^
